# In the eyes of the beholder: investigating the effect of visual probing on accuracy and gaze fixations when attending to facial expressions among primary and secondary callous-unemotional variants

**DOI:** 10.1007/s00787-019-01452-z

**Published:** 2019-12-06

**Authors:** Melina Nicole Kyranides, Kostas A. Fanti, Maria Petridou, Eva R. Kimonis

**Affiliations:** 1 Department of Clinical and Health Psychology, School of Health in Social Science, Medical School (Doorway 6), University of Edinburgh, Teviot Place, Edinburgh, EH8 9AG United Kingdom; 2 Department of Psychology, University of Cyprus, Nicosia, Cyprus; 3 Department of Psychology, University of New South Wales, Sydney, Australia

**Keywords:** Eye tracking, Callous-unemotional traits, Primary psychopathy, Secondary psychopathy, Facial emotion recognition

## Abstract

Individuals with callous-unemotional (CU) traits show deficits in facial emotion recognition. According to preliminary research, this impairment may be due to attentional neglect to peoples’ eyes when evaluating emotionally expressive faces. However, it is unknown whether this atypical processing pattern is unique to established variants of CU traits or modifiable with intervention. This study examined facial affect recognition and gaze patterns among individuals (*N *= 80; *M* age = 19.95, SD = 1.01 years; 50% female) with primary vs secondary CU variants. These groups were identified based on repeated measurements of conduct problems, CU traits, and anxiety assessed in adolescence and adulthood. Accuracy and number of fixations on areas of interest (forehead, eyes, and mouth) while viewing six dynamic emotions were assessed. A visual probe was used to direct attention to various parts of the face. Individuals with primary and secondary CU traits were less accurate than controls in recognizing facial expressions across all emotions. Those identified in the low-anxious primary-CU group showed reduced overall fixations to fearful and painful facial expressions compared to those in the high-anxious secondary-CU group. This difference was not specific to a region of the face (i.e. eyes or mouth). Findings point to the importance of investigating both accuracy and eye gaze fixations, since individuals in the primary and secondary groups were only differentiated in the way they attended to specific facial expression. These findings have implications for differentiated interventions focused on improving facial emotion recognition with regard to attending and correctly identifying emotions.

**Electronic supplementary material:**

The online version of this article (10.1007/s00787-019-01452-z) contains supplementary material, which is available to authorized users.

## Introduction

Accurately processing emotional expressions is critical in everyday functioning and social interactions. Deficits in facial affect recognition found in individuals with psychopathic traits [1] and conduct problems (CP [2, 3] are thought to explain their greater engagement in antisocial behavior. Several studies show that callous-unemotional (CU) traits, the putative childhood antecedent to psychopathy that is characterized by remorselessness and lack of empathy and concern for others’ distress, are also associated with facial affect recognition deficits [1, 4]. Findings suggest that reduced attention to faces, especially to the eye region, underlie emotion recognition difficulties among youth with CU traits [4, 5]. Despite the need to understand the developmental continuity of these problems, no prior work has investigated such processing deficits by monitoring participants’ stable CU traits and antisocial behavior across adolescence and young adulthood. Further, few studies consider well established heterogeneity in psychopathic traits. In other words, co-occurring anxiety levels phenotypically distinguish between emotionally distinct primary and secondary subtypes of psychopathy and CU traits [6, 7]. The present study examines whether facial affect recognition and associated eye gaze patterns differentiate young adults identified with primary-low anxious and secondary-high anxious CU traits, and whether these patterns can be manipulated by re-directing attention to various parts of the face.

## Heterogeneity in CU traits

There is increasing empirical support for heterogeneity among community and incarcerated adolescents and adults characterized by CU traits, with a number of studies focusing on the primary and secondary distinction [6–9]. According to prior work [10, 11], primary psychopathy arises as a function of genetically-based deficits in emotion processing that might lead to lack of anxiety. In contrast, secondary psychopathic traits or acquired callousness might develop due to environmental influences, such as parental maltreatment or traumatic exposure, resulting in poorly regulated negative affect characterized by high anxiety, emotional distress, and impulsivity [8, 12, 13]. Understanding the developmental mechanisms underlying these distinct psychopathic profiles could lead to improved interventions as this population tends to be less-responsive to treatment efforts [14].

## Psychopathic traits and facial expression recognition

Face recognition is a basic component of interpersonal communication and paying attention to these non-verbal cues enables the viewer to infer the thoughts, emotional state and intentions of others [15]. If social cues are not attended to or incorrectly recognized they will misguide the observer from understanding the intention and actions that might follow. Several theories of psychopathy suggest that individuals with psychopathic traits process emotions differently than typically developing individuals, which is thought to contribute to their development of persistent antisocial behavior [16]. For example, Blair’s [16] violence inhibition mechanism (VIM) posits that individuals with psychopathic traits fail to experience the fear and sadness of others as aversive, leading to greater engagement in antisocial behavior.

There is robust evidence that adults [17, 18] and children with psychopathic and CU traits [4, 5, 9] show facial affect recognition impairments. However, there is controversy over whether this impairment is a general recognition deficit for all emotions [19, 20] or specifically in response to others’ fear [2, 4, 5, 21] or sadness [22, 23]. In line with this idea, Martin-Key and colleagues [2] found that higher levels of CP alone and CU-traits alone were linked to reduced fear recognition, whereas interactive effect between CP and CU-traits was associated with better fear recognition. This inconsistency in findings might reflect a failure to consider heterogeneity among individuals with psychopathic traits as preliminary research suggests that primary and secondary CU variants differ in their facial affect recognition and emotional attention processing [9, 22, 23]. A recent study conducted with children with behavioral problems [9] taking anxiety into account found that emotion recognition deficits were more characteristic of primary CU traits. In contrast, Gillespie and colleagues [23] found no relationship between primary and secondary psychopathic traits and emotion recognition accuracy. However, the study conducted by Gillespie and colleagues [23] used a sample of young adults and did not take anxiety into account.

## Psychopathic traits and eye gaze deficits

The precise mechanism underlying the impairments in emotion recognition found in individuals with psychopathic traits remains unclear. Functional neuroimaging studies with children high on CU traits have revealed hypoactivity of the amygdala, a crucial structure for processing fearful [24] and sad [25] facial expressions. These results suggest that impairments in recognition of distressing emotions among individuals with psychopathic traits may reflect amygdala dysfunction. Individuals with psychopathic traits and patients with amygdala damage fail to fixate on the eye region when processing facial expressions, which is essential for recognizing fear [26]. Thus, reduced attention to the eye region when processing facial expressions may be a critical mechanism in the development of psychopathic traits. Consistent with this hypothesis, most eye tracking studies found that CU traits were associated with fewer and shorter fixations to the eye region during a facial expression recognition task in fearful stimuli [5], yet others found reduced fixations for surprised faces [2]. Similarly, primary psychopathic traits were associated with a reduced number of fixations and lower overall dwell time on the eyes relative to the mouth across different facial expressions in an adult community male sample [23]. Further, adolescents with primary CU traits showed deficits in orienting to others’ distress cues whereas those with high-anxious secondary CU traits were hypervigilant to these stimuli [7]. An amygdala deficit might underpin the inability of individuals with psychopathic traits to orient their attention to emotional cues [27].

Offering promise for intervention efforts, there is evidence that deficits in emotion recognition can be temporarily corrected. To illustrate, prior work found that a simple verbal instruction to fixate on the eye region of emotional faces led to enhance facial emotion recognition accuracy in children with antisocial behavior and CU traits [4, 5], and in patients with amygdala dysfunction [26]. Thus, a key goal of the present study was to examine whether a visual probe (acting more as a silent probe) randomly appearing to redirect participant’s attention to various parts of the face, and not only the eye region, could improve emotion recognition, in low-anxiety-primary and high-anxiety-secondary CU subtypes and controls.

## Current study

The main objective of the current study was to examine how primary and secondary CU sub-groups and controls process emotional faces by examining their (a) emotion recognition accuracy and (b) eye gaze patterns using eye tracking. A second aim of this study was to test the influence of a visual probe designed to re-direct attention to specific facial regions. This study aimed to improve upon prior research in three key ways. First, subtypes are identified based on longitudinal assessments of CP, CU traits and anxiety levels at two different developmental periods (adolescence and adulthood), contrasting against the majority of prior research that relied on a single self-report measure at a single time point, which may yield unstable estimates. Screening assessments were based on a large community sample and data from at risk participants were collected during adolescence, who were reassessed again during adulthood. These stable scores were used to distinguish primary (high scores on CP, CU traits and low scores on anxiety) and secondary (high scores on CP, CU traits and anxiety) variants and Controls (low scores across measures). Second, the effect of re-directed attention to three main regions (forehead, eyes, mouth) of interest using a visual cue was assessed using eye-tracking techniques to measure total number of fixations in each region, relative to prior studies that only focused on the eye and/or mouth regions [5, 23]. Third, we used dynamic stimuli of six facial expressions including angry, fearful, painful, sad, happy and neutral facial expressions. Dynamic facial expressions have a higher ecological validity over static stimuli [25, 28, 29] which is critical since scanning facial expressions can vary depending on the type of emotional expression [5, 30].

Based on theoretical suggestions that psychopathic traits are linked with reduced attention to the eyes of emotional faces [4, 5, 23], it was hypothesized that individuals with either primary and secondary CU traits would show reduced accuracy when processing facial expressions compared to controls, in agreement with prior research [1, 4, 5]. Furthermore, individuals in the primary CU group were expected to perform worse on the facial recognition task compared to the secondary CU group as suggested by a recent study [9]. Regarding eye-gaze patterns, it was hypothesized that individuals in both primary and secondary CU groups would show fewer fixations to the eye-region of the face relative to controls, and these deficits were expected to be specific to distress cues of fear, sadness, and pain [5, 23, 24]. Due to their high levels of anxiety and hypervigilance to affective stimuli, the high anxious secondary group was expected to show a higher number of fixations to threatening stimuli (i.e., facial expressions of anger) compared to the low-anxious primary group, suggesting attention bias to threat. This suggestion is based on cognitive accounts of anxiety, which suggest that anxious individuals show a preferential tendency to allocate attention to threat (i.e., others’ anger and hostility [31]). Additionally, if the impairment in facial affect recognition is due to reduced attention to the eye region when identifying facial expressions for individuals with CU traits, research suggests this can be temporarily reversed [4, 5].Thus, it was hypothesized that the visual probe directing participants’ attention to the eye region of the face would increase accuracy ratings for individuals with psychopathic profiles, and in particular for individuals in the primary CU group especially when presented with fearful, sad and painful expressions. Due to lack of research, it is not clear whether the probe manipulation directing attention to the eye region would be effective in increasing accuracy ratings across all emotions, and as a result no specific predictions were made for other emotions.

## Method

### Participants and screening

Data were collected from high schools in three different provinces in Cyprus (Nicosia, Limassol and Larnaca) at three different time points as part of a longitudinal investigation of the development of psychopathological problems [6, 32]. The first two screening assessments took place when participants were approximately 16 (*N* = 2414; *M* age = 15.96, SD = 0.89 years; 55% female) and 17 (*N *= 2306; *M* age = 16.99, SD = 0.91 years; 52% female) years old, 6 months apart. Participants differing on levels of CP, CU traits, and anxiety across two waves of data collection were selected to participate in the experimental phase of the study if they presented both high CP and CU traits in adolescence (time 1 and 2) but differed in levels of anxiety. Participants scoring significantly higher on CU traits and CP, but not anxiety (compared to controls) formed the primary group and participants scoring significantly higher on anxiety, CU traits and CP (compared to controls) formed the secondary group (see Table 1 for group comparisons). Participants scoring low on all measures of CP, CU traits and anxiety formed the control group. The third assessment took place approximately 3 years later.

**Table 1:** Comparisons between identified groups for the study sample

Variable	Primary (*n* = 26)	Secondary (*n* = 19)	Controls (*n* = 35)	*F* value	*df*	*η*^2^
CU traits						
ICU time 1	30.84 (2.01)^b^	29.07 (2.34)^b^	17.25 (1.66)^a^	16.45*	2	0.36
ICU time 2	31.37 (2.02)^b^	28.57 (2.36)^b^	17.32 (1.67)^a^	16.51*	2	0.36
ICU time 3	29.47 (1.97)^b^	28.14 (2.29)^b^	14.79 (1.62)^a^	20.65*	2	0.41
Conduct problems						
YI-4 time 1	6.05 (1.16)^b^	9.71 (1.35)^c^	2.11 (0.95)^a^	11.13*	2	0.28
YI-4 time 2	9.00 (1.47)^b^	12.64 (1.71)^b^	1.75 (1.21)^a^	15.60*	2	0.35
YI-4 time 3	6.05 (0.92)^b^	6.50 (1.07)^b^	2.25 (0.76)^a^	7.60*	2	0.21
Anxiety						
YI-4 time 1	4.47 (0.77)^a^	11.71 (0.90)^b^	4.60 (0.63)^a^	24.60*	2	0.46
YI-4 time 2	5.10 (0.68)^a^	10.36 (0.79)^b^	5.82 (0.56)^a^	14.58*	2	0.34
STAI time 3	44.95 (1.27)^a^	52.79 (1.48)^b^	43.00 (1.04)^a^	15.04*	2	0.34

Estimated marginal means (SE). Different subscripts (a, b, c) denote significant differences between groups in post hoc pairwise comparisons

*CU* callous unemotional traits, *ICU* inventory of callous unemotional traits, *YI-4* Youth’s Inventory, *STAI* State-Trait Anxiety Inventory

*p < 0.01

Ninety participants (*M* age = 19.90, SD = 0.99 years) were originally selected on the basis of their baseline scores on measures of CU traits, CP and anxiety collected during adolescence and were contacted to participate in a follow up assessment and experimental session, when participants were young adults. All 90 participants contacted consented to participate in a follow-up session that involved completing questionnaires administered using a secure internet platform. Eighty-two of those participants (91% of the participants contacted) also agreed to participate in an experimental session. Participants that declined to participate in the experimental session did not differ from those who participated in the experiment with regard to their baseline measures of CU traits, CP, Anxiety or sex (all *p*s> 0.05). Data from two participants were omitted from analyses due to equipment malfunction, leaving 80 participants that comprised the final study sample (*M* age = 19.95, SD = 1.01 years; 50% female). These participants, initially identified based on their scores on CU traits, CP, and anxiety assessed during the first two waves of measurement in adolescence, continued being differentiated as expected during the third wave of measurement (Table 1). These findings point to continuity in levels of CU traits, CP, and anxiety and validate the identified groups. Participants scoring continuously high on CP, CU traits and anxiety represented the secondary CU group (*n *= 19; 42% female), while participants with high CP and CU traits but low anxiety scores across time represented the primary CU group (*n *= 26, 46% female). The control group was represented by participants scoring low on all measures (*n *= 35, 57% female). These differences are presented in “Results”.

### Measures

The Inventory of Callous-Unemotional Traits (ICU [33]) was used to measure CU traits at all assessment points. The ICU is a self-report scale consisting of 24 items (e.g., “I do not feel remorseful when I do something wrong”) that are rated on a 4-point scale ranging from 0 (*not at all*) to 3 (*definitely true*), with higher scores indicating greater CU traits. In the present study, ICU scores demonstrated adequate internal consistency across all three assessment points (*α*_1_ = 0.77, *α*_2_ = 0.80, *α*_3_ = 0.89). The reliability and construct validity of the Greek version of the ICU that have been supported by prior work [32, 34].

The Youth’s Inventory-4 (YI-4 [35]) was used to measure CP at all three assessment points and Anxiety at two assessment points during adolescence. The YI-4 is a self-report rating scale designed to assess DSM-IV symptoms of emotional and behavioral disorders in adolescents, which is supported for use with young adults [32]. Participants rate YI-4 symptoms on a 4-point scale of 0 (*never*) to 3 (*very often*). Only items from the Conduct Disorder (15 items; e.g. “I break into houses, buildings, or cars”) and Anxiety (6 items; e.g. “I have trouble getting myself to stop worrying”) subscales were used. In the present study, CP scores demonstrated adequate internal consistency at all three time points (*α*_1_ = 0.90, *α*_2_ = 0.91, *α*_3_ = 0.84). Anxiety scores also demonstrated adequate internal consistency in the current sample across the two assessment points (*α*_1_ = 0.85, *α*_2_ = 0.83). Previous research has supported the validity of YI-4 scores in community and clinical samples in the United States and Cyprus [36, 37].

The State-Trait Anxiety Inventory (STAI-T [38]) is a 40-item self-report measure that indexes the intensity of state and trait anxiety. Items are scored using a 4-point Likert scale ranging from 1 (*almost never*) to 4 (*almost always*). In the current study, only items assessing trait anxiety (20 items; *α* = 0.74) were used. This scale captures an individual’s general tendency to perceive situations as threatening. STAI-T scores have demonstrated acceptable internal reliability and construct validity in prior research [37].

## Experimental materials

Facial Emotion Recognition was assessed in the experimental session by having participants view a series (*n *= 48) of standardized stimuli of dynamic visual stimuli (clips) of facial expressions from the Montréal Pain and Affective Face Clips (MPAFC) database [28, 29]. Facial expressions from four female and four male adults expressing one of six expressions: anger, fear, happiness, sadness, pain and neutral, were presented in pseudo-randomized order to avoid sequential repetition, resulting in 48 trials. Each trial consisted of three sequential and non-overlapping components: (1) 1-second fixation cross appearing in the center of the screen, (2) 1-second asterisk (i.e. probe), and (3) 1-second presentation of the dynamic facial expression. After the presentation of each stimulus, participants were asked to identify the emotion being expressed (happy, sad, angry, fear, pain or neutral) by logging their response via key press. Participants were instructed to respond as quickly and as accurately as possible. No instructions were provided to participants regarding the probe, and as a result the probe acted as a silent cue. Specifically, to examine if participants’ ability to recognize the various facial expressions could be modified (enhanced or impaired), during each facial stimulus an asterisk appeared at one of the following locations: (a) the top part of the face around the forehead; (b) the center of the face around the eyes; (c) at the lower part of the face, around the mouth. The distance between the probes appearing at the eyes and the forehead was the same as the distance between the probes appearing at the eyes and mouth. The task took 15–20 min to complete and participants were informed of the study objectives after the completion of the experiment.

**Fig. 1:**
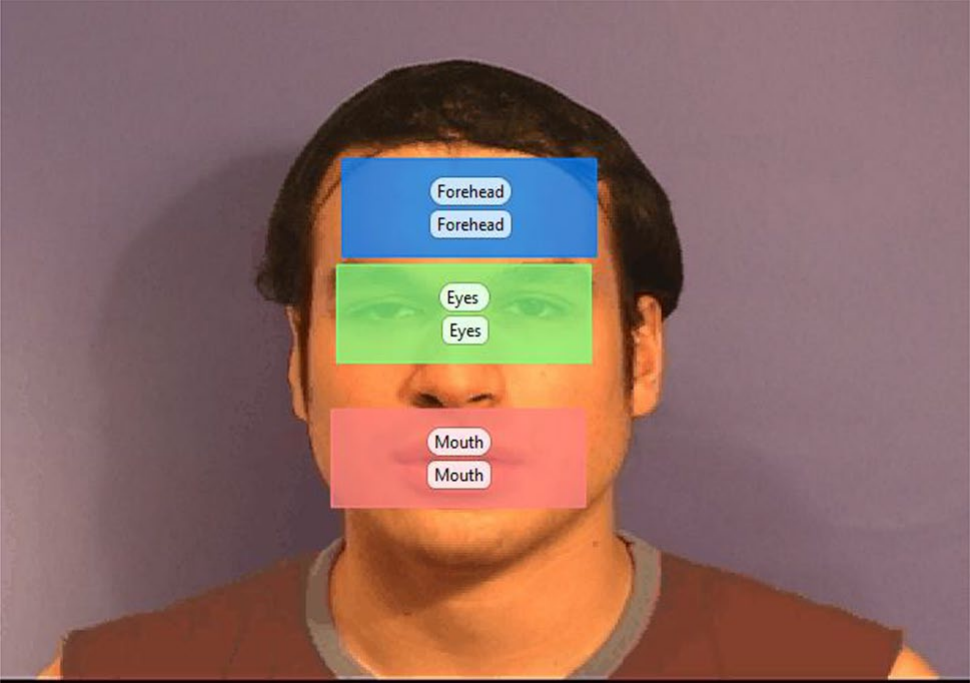
Areas of interest for facial expression recognition task

Real-Time Attention Allocation was assessed during the facial emotion recognition task using Tobii X120 eye-tracking equipment and software (Tobii Technology, Inc, Washington, USA). The Tobii X120 has an accuracy of 0.5°, a drift that is less than 0.3° and a sampling frequency of 120 Hz. Pupil locations can be mapped to gaze locations on the screen by a 5-point calibrating system. Tobii Studio 3.0.1 (Tobii Technology, Inc, Washington, USA) was used for the timing of events, presentation of visual stimuli and recording eye movements. Three equally sized regions of interest consistent across all face stimuli were defined for the forehead, eye and mouth regions (see Fig. 1). Measures included eye-gaze visits in the pre-determined areas of interest, and correct responses. In the 1 s presentation of the stimuli, the number of fixations ranged from 0 to 7 for angry, sad and painful facial expressions, 0–8 for fearful, and 0–6 for happy and neutral facial expressions. The average number of fixations for each facial expression for the groups are presented in Fig. 3.

## Experimental procedure

After providing informed consent, participants were seated in a height-adjustable chair, which was adjusted to the point at which their gaze was most accurately recorded. The chair was placed approximately 60 cm from a computer screen (47 × 24.5 cm). The size of the stimuli was 20 × 14.5 cm, which translates to visual angles of approximately 7°–10°. A calibration test was performed before administering the face recognition task in order to check that eye gaze was recorded correctly. Following the calibration test, participants were administered the task. All participants were debriefed and provided financial compensation (€15) for their participation.

### Plan of analysis

The analyses had three main objectives: (1) Group differences: analysis of variance (ANOVA) was used to test whether the groups identified during adolescence and adulthood were differentiated on measures of CU traits, CP and anxiety assessed at the three time points. (2) Emotion recognition accuracy: a repeated measures ANOVA was conducted in SPSS 24 with groups (primary, secondary, control) as the between subjects variable and accuracy for the six facial expressions (anger, fear, sadness, pain, happiness, neutral) and probe location (up, down, center) as within subjects variables. (3) Fixations: repeated measures ANOVAs were conducted with group (primary, secondary, control) as the between subjects variable and the three predetermined facial areas of interest (eyes, forehead, mouth), probe location (up, down, center) and six emotions (anger, fear, sadness, pain, happiness and neutral) as within subjects variables. The analysis was conducted for total fixations within the predetermined areas of interest for the entire presentation period. It was assumed that fixation count reflects a combination of participants’ interest and attraction to the pre-determined areas of interest.

## Results

### Group differences

Results from the ANOVA comparing the three identified groups on CU traits, CP and anxiety during the three waves of measurement are reported in Table 1. Significant differences on levels of CU traits, CP, and anxiety were identified across all assessment points. Post hoc comparisons indicated that the mean scores for CU traits were significantly higher for individuals in the primary and secondary groups, compared to controls on all three assessment points. Regarding levels of CP, post hoc comparisons indicated that during time 1 participants in the secondary group scored higher compared to the primary (*p *= 0.04) group, and both the primary and secondary groups scored higher compared to controls (*p*s < 0.01). During times 2 and 3, primary and secondary groups scored similarly on CP, and significantly higher than controls. Finally, individuals in the secondary group scored higher than both other groups on anxiety across all assessment points.

### Facial emotion recognition accuracy

The repeated measures ANOVA examining differences in correctly categorizing facial expressions revealed a significant effect for groups predicting average levels of accuracy (i.e., across all six facial expressions), *F*(2,77) = 5.15, *p* < 0.01, *η*^2^= 0.12, with controls showing higher overall accuracy (*M *= 90.80, SE = 1.50) compared to both the primary (*M *= 86.10, SE = 1.70) and secondary (*M *= 83.20, SE = 2.00) CU groups (*p *= 0.04 and *p *< 0.01 respectively). Post hoc comparisons indicated no significant differences between primary and secondary CU variants in accuracy ratings (*p *= 0.27).

There were also two significant within group effects. Firstly, in relation to emotion accuracy, *F*(5,385) = 19.77, *p *< 0.001, *η*^2^= 0.20, participants were most accurate at identifying happy (*M *= 95.00, SE = 1.10) followed by neutral (*M *= 91.00, SE = 1.50), fearful (*M *= 89.90, SE = 1.60), sad (*M *= 87.00, SE = 1.90), angry (*M *= 83.40, SE = 1.70) and pain (*M *= 73.80, SE = 2.70) facial expressions. Post hoc comparisons indicated that participants were significantly more accurate at correctly identifying happy expressions, whereas they were less accurate in identifying pain expressions compared to all other facial expressions (all *p*s < 0.05), which is aligned with prior work [39]. Secondly, results showed a significant within group effect for probe location, *F*(2,154) = 13.09, *p *< 0.001, *η*^2^= 0.15. Post hoc comparisons indicated that participants’ accuracy was higher when the probe appeared in the lower part of the face (*M *= 90.90, SE = 1.20) relative to the two other probe locations (both *p*s< 0.001), across emotion conditions. Accuracy was similar when the probe appeared at the center of the face (*M *= 84.90, SE = 1.40) relative to the top part of the face (*M *= 84.20, SE = 1.30; *p *= 0.65).

**Fig. 2:**
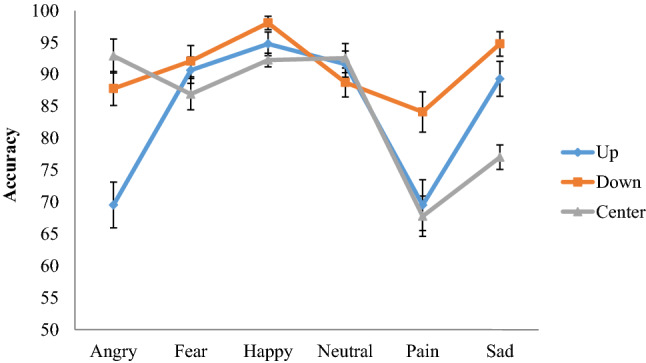
Emotion × probe location interaction with accuracy as the dependent variable

Finally, there was a significant interaction effect between the emotion expressed and probe location, *F*(10,770) = 8.44, *p *< 0.001, *η*^2^= 0.10. As illustrated in Fig. 2, participants were less accurate during angry expressions when the probe appeared at the top part compared to the center or the bottom of the face. In contrast, participants were less accurate in rating sad expressions when the probe appeared at the center compared to the top or the lower part of the face. For pain expressions, participants where more accurate when the probe appeared at the bottom of the face compared to when the probe appeared at the top or center parts of the face (all *p*s < 0.05). No significant interactions between groups with accuracy and probe location were identified.

A series of additional *t* tests comparing the first half of trials to the second half of the trials were conducted to examine practice effects for each emotion and condition. Accuracy was higher for the second trials compared to the first set for the following facial expressions and conditions: angry when the probe was displayed at the top and lower part of the face, pain when the probe was displayed at the top and middle of the face and neutral facial expression when the probe was presented at to top part of the face (all *p*s < 0.05). No other differences were identified.

### Fixation count

The repeated measures ANOVA predicting fixation count did not identify a main effect of groups, *F*(2,78) = 1.78, *p *= 0.18, *η*^2^= 0.04. Within group effects for predetermined areas of interest, *F*(2,154) = 54.17, *p *< 0.001, *η*^2^= 0.41, probe location, *F*(2,154) = 12.80, *p *< 0.001, *η*^2^= 0.14, and emotion, *F*(5,385) = 4.36*, p *< 0.01, *η*^2^= 0.05, were identified. Findings suggested that participants visited the mouth (*M *= 2.29, SE = 0.15) and eye regions (*M *= 2.24, SE = 0.13) more frequently than the forehead (*M *= 0.82, SE = 0.07) area (both *p*s< 0.001). The number of fixations in the mouth and eye region were similar (*p *= 0.80). Further, participants’ overall fixations on all predetermined areas of interest increased when the probe appeared at the top part of the face (*M *= 1.91, SE = 0.09) compared to when it appeared at the bottom (*M *= 1.72, SE = 0.08; *p *< 0.001) or the center of the face (*M *= 1.71, SE = 0.08; *p *< 0.001). The difference between number of fixations when the probe appeared at the center or and lower part of the face were not significant (*p *= 0.86). Finally, fixation counts were less for sad expressions compared to fear, happy, and pain (*p*s< 0.05).

**Fig. 3:**
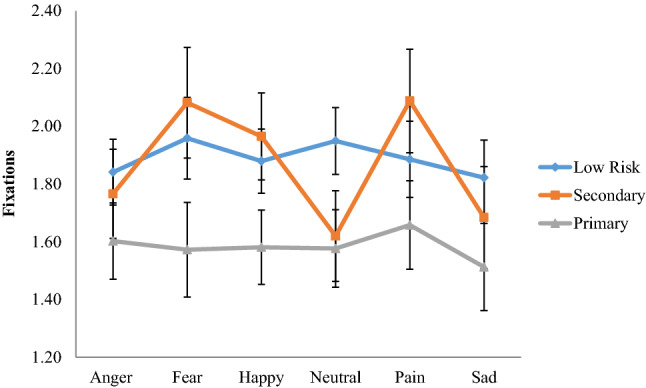
Emotion × group interaction with fixation count to the face (all areas) as the dependent variable

The analysis also revealed a significant emotion × group interaction, *F*(10,385) = 2.32, *p *< 0.05, *η*^2^= 0.06. As illustrated in Fig. 3, pairwise comparisons between groups broken down by emotions suggested that individuals with primary CU traits showed fewer overall fixations when attending to *angry* facial expressions (*M *= 1.60, SE = 0.13), compared to the secondary (*M *= 1.68, SE = 0.18; Cohen’s *d *=0.25) and control (*M *= 1.84, SE = 0.11; *d  *= 0.36) groups but none of these differences were significant (all *p*s > 0.05). Pairwise comparisons between groups for *fearful* expressions showed that individuals with primary CU traits showed fewer overall fixations (*M *= 1.57, SE = 0.16) compared to those in the secondary group (*M *= 2.08, SE =  0.19; *d *=0.62; *p *< 0.05) but the difference with the control group (*M *= 1.96, SE = 0.14; *d *=0.47) approached significant levels (*p *= 0.07). When viewing *happy* faces, the fixations were fewer for the primary group (*M *= 1.58, SE = 0.13) compared to controls (*M *= 1.88, SE = 0.11; *d *=0.46) and the secondary group (*M *= 1.97, SE = 0.15; *d *=0.60), but only the difference between the primary and secondary group approached significance (*p * = 0.06). The difference in fixations between the secondary group and controls for angry, fearful and happy faces was of small effect (*d *= 0.11–0.15; all *p*s > 0.05). For *neutral* facial expressions, individuals with primary CU traits displayed (*M *= 1.58, SE = 0.13) similar fixations to individuals with secondary CU traits (*M *= 1.62, SE = 0.16; *d  *= 0.06; *p  *= 0.83). Both primary and secondary groups showed less fixations compared to controls (*M *= 1.95, SE = 0.12) (*d  *= 0.55; *p *< 0.05 and *d *=0.45; *p *< 0.05 respectively). When viewing *painful* facial expressions, the primary group (*M *= 1.66, SE = 0.15) showed fewer overall fixations compared to controls (*M  *= 1.89, SE = 0.13; *d * = 0.30; *p * = 0.16) and the secondary group (*M *= 2.09, SE = 0.18; *d * = 0.56; *p *< 0.05). The difference in fixations displayed by the control group and the secondary group when viewing painful expressions was small and not significant (*d  *= 0.26; *p * = 0.27). Finally when viewing *sad* facial expressions the primary group showed fewer overall fixations (*M *= 1.51, SE = 0.15) compared to the secondary group (*M *= 1.68, SE = 0.18; *d  *= 0.23) and controls (*M *= 1.82, SE = 0.13; *d *=0.41), but none of these differences were significant (all *p*s> 0.05).

Results of repeated measures ANOVA also revealed several significant interactions not involving the identified groups. The following two way interactions were identified: emotion X area of interest, *F*(10,770) = 11.11, *p *< 0.001, *η*^2^= 0.13, emotion × probe location *F*(10,770) = 6.34, *p *< 0.001, *η*^2^= 0.08, and probe location × area of interest interactions, *F*(4,308) = 33.79, *p *< 0.001, *η*^2^= 0.31. These interactions were represented in a higher order three-way interaction of emotion, area of interest and probe location *F*(20,1540) = 3.50, *p *< 0.001, *η*^2^= 0.04. Post-hoc analysis probing this higher order interaction showed that attention to various areas of the face was more pronounced for particular emotions, although these effects depended on probe location (see supplementary material SM1). Across the majority of emotions and irrespective of probe location (except fear when the probe was up), fixation counts in the forehead area were less compared to the eyes and mouth. When the probe appeared at the top part of the face, more fixations were directed to the eyes than the mouth for fearful and angry facial expressions. When the probe was located at the centre or the bottom of the facial expression, more fixations were directed to the mouth than the eyes, during happy facial expressions. Finally, when the probe appeared at the lower part of the face, more fixations were displayed in the mouth area compared to the eyes, for pain and sad emotions.

## Discussion

The present study examined how individuals with stable primary and secondary CU traits, relative to each other and to controls, categorize and attend to facial expressions by examining their accuracy ratings and eye gaze fixations. It also investigated the effect of a visual cue used to direct participants’ attention to different parts of the face, across groups. Overall, individuals in both primary and secondary CU groups were less accurate than controls at recognizing facial expressions across multiple emotions. When considering the eye fixation data, however, the primary group showed fewer overall fixations compared to the secondary group for fearful and painful facial expressions. These findings support heterogeneity in the construct of CU traits, whereby individuals high on anxiety differed from those low on anxiety in how they attended to fearful, and painful facial expressions, which might stem from their differences in anxiety. We failed to find that individuals with primary or secondary CU traits showed fewer fixations to the eye region specifically when processing facial expressions [5, 40]. Instead, all participants, regardless of levels of CU traits most frequently attended to the mouth and eye regions of the face when processing facial expressions, compared to less visited forehead area, consistent with prior research [39, 41]. The inconsistency in findings may be attributed to methodological differences between studies in task parameters (i.e., verbal cue vs visual cue, static vs. dynamic facial stimuli) and group formation, discussed in greater detail below.

In support of our main hypothesis, we found that individuals with primary and secondary groups were less accurate than controls across all emotional conditions but did not differ from each other. This finding is in line with prior work [2, 42]. More specifically, Prado and colleagues [42] found in a non-clinical sample that both primary and secondary psychopathy subtypes showed reduced accuracy in identifying facial affect, although they found that the primary group showed more profound impairments. The inconsistency in findings between our study and Prado and colleagues [42] could be attributed to methodological differences in the identification of the groups. More specifically Prado and colleagues [42], used the Levenson self-report measure at a single time point to identify primary and secondary groups, whereas we used a combination of measures of conduct problems, callous unemotional traits and anxiety, assessed over time. As indicated by the accuracy ratings, our findings also suggest that this impairment in facial affect recognition for individuals high on CU traits is more generalized, in line with meta-analytic findings [19] and a study conducted with adolescents with CP (with and without anxiety); [3] that emotion recognition deficits in psychopathy are not restricted to specific emotions such as fear or sadness as has been suggested by prior work [5, 17, 43]. The stimuli used in the current study were dynamic snapshots (clips) of adults expressing various emotions and included movements in different parts of the face compared to static stimuli that were used in other studies [5, 24] possibly contributing to the differences in results.

The emotion recognition impairment found in primary and secondary CU variants was associated with divergent attention patterns, as indicated by eye gaze fixations to fearful and painful facial expressions. Specifically, both primary and secondary groups showed fewer overall fixations when viewing neutral faces compared to controls, suggesting that lack of attention to the face is a problem in individuals with CU traits when attending to neutral or more subtle facial expressions, irrespective of their anxiety levels. It is possible that neutral expressions do not capture the attention of individuals high on CU traits in the same way as typically developing individuals. Importantly, distinct eye-gaze fixation differences were identified for specific emotions, with the primary group showing less attention to fearful and painful faces compared to the secondary CU group. These results suggest that anxiety levels in individuals with CU traits might account for subtype differences in attention to fearful and painful expressions. It is possible that low-anxious individuals with primary CU traits are less engaged by the fearful and painful expressions (avoid, ignore), whereas high-anxious individuals with secondary CU traits have stronger attention bias to negative emotions, which is reflected by an increase in fixations to the fear and pain of others. This finding is in accordance to prior work showing that anxiety is associated with deficits in attending to threating stimuli, suggesting that it might be the combination of anxiety and CU traits that drive these differences [31].

Although fewer fixations to the eye region of the face has been proposed as a mechanism explaining impaired empathetic processing in individual with psychopathic traits [2, 5, 40, 44], we failed to find that individuals with primary or secondary CU traits showed less fixations to the eye region specifically when attending to facial expressions, relative to controls. Instead, all participants, regardless of level of CU traits, attended to the mouth and eye regions more frequently when processing facial expressions, compared to less important areas of the face such as the forehead [45]. It should be noted, however, that in the current study (a) we used a visual probe to direct participants’ attention to various parts of the face, (b) we examined areas of the face which are less visited (forehead) and (c) defined the areas of interest differently compared to other studies [5, 44]. The face stimuli used were not static, were displayed for less time (compared to other studies e.g. [44] and the areas of interest were divided into three equally defined areas, which overall covered the face. In addition, more recent studies using clinical populations with psychopathic traits [40, 44] found that reduced attention to the eyes of the face was related to the interpersonal dimension of psychopathy, which was not assessed in the current study.

Unexpectedly, the visual cue that was used to redirect participants’ attention to various parts of the face enhanced accuracy ratings for all emotions when it appeared at the lower part of the face, and not when specifically directing attention to the eye region. An unexpected finding was that when the probe appeared at the eye level, this negatively impacted accuracy ratings only for pain and sad facial expressions compared to when the probe appeared in the other two locations (forehead and mouth), for all participants. A possible explanation is that pained and sad facial expressions involved movement in the lower part of the face, so when the probe directed participants’ attention away from the mouth region this might have distracted participants and detrimentally affected their accuracy ratings. Contrary to predictions, the visual cue manipulation used in the current study was not beneficial in improving fear recognition specifically for individuals with CU traits [5]. Nevertheless, findings suggest that such probe manipulations can be effective in directing participants’ attention to different parts of the face but can also work as a distractor depending on the emotion being displayed. The current findings can inform intervention efforts focused on improving emotion recognition. There is evidence suggesting that by improving emotion recognition in young offenders this can lead to a reduction in the severity of criminal acts [46] which suggests that the ability to recognize emotions has important consequences for behavior. Developing separate training programs that focus on emotion recognition difficulties in individuals with behavioral problems and CU traits that target underlying attention mechanisms and attentional biases are crucial.

The above findings should be interpreted with caution and viewed within the context of some limitations. First, this study was conducted with a community sample with at risk individuals and results need to be replicated in clinical and forensic samples with higher levels of psychopathic/CU traits before they can be generalized. Additionally, this study did not include a high CU group with low CP to examine if the effects are driven by CU or CP, which can be explored in future studies which could also examine different sub-dimensions of psychopathy. Second, the ceiling effects for participants’ performance on the facial task indicated that it was not very challenging for some emotions (e.g. happy facial expressions); thus, a more sensitive measure is warranted for future investigations. Third, given the brief presentation of stimuli, participants did not have the opportunity to fixate on many parts of the face, which might have resulted in reduced fixation counts across emotions and participant groups. Additionally, based on the data collected in this study we were not able to further analyze the location of first and second fixation that would allow us to better understand the eye gaze patters of the two groups. Nevertheless, the overall group effects found are suggestive of distinct patterns of attention allocation between individuals with primary and secondary CU traits when deciphering fearful and painful expressions and are suggestive of potentially distinct mechanisms underlying their shared CU phenotypes. These findings are aligned with recent studies [47, 48] showing emotion recognition deficits in distress cue expressions are due to a shared genetic diathesis common to both distress cue recognition and CU traits. Although these studies did not examine the primary-secondary distinction, future research should replicate these findings using similar groups.

Despite the aforementioned limitations, the current study contributes to the literature in a number of ways. This is the first study to examine emotion processing differences between primary and secondary CU groups identified on the basis of assessments of anxiety, CU traits and CP, across two different developmental periods. The fact that individuals with primary and secondary CU traits both show impairments in facial affect recognition but show differences in attending to fearful and painful expressions is relevant for empathy interventions. This is also the first study to use a visual cue to direct participants’ attention to various parts of the face, and to assess the effectiveness of this manipulation by examining accuracy ratings and eye fixations. Directing attention to the eye region of the face does not always have the desired effect of improving accuracy for all emotion conditions and appears more complex. The replicability of these findings with regard to the manipulation used should be examined in additional research.

Finally, the current study has a number of clinical implications. Considering anxiety levels has been useful in identifying individuals with different CU subtypes. Both primary and secondary CU groups show deficits in processing facial emotional expressions; however, the two groups differ in how they attend to these stimuli. These findings have the power to inform future intervention efforts. More specifically, the data suggest that cognitive retraining approaches such as attention bias modification [31, 49] allow us to address such biases by redirect attention to salient disorder-specific cues. For individuals with primary CU traits whose recognition impairments appear to stem from deficient fixation on face stimuli (avoidance) the target in attention training should to be to increase attention to treat-related stimuli. In contrast for individuals with secondary CU traits the attention training should focus on redirecting attention away for treat related stimuli.

## Electronic supplementary material

Below is the link to the electronic supplementary material.

Supplementary material 1 (DOCX 19 kb)
