# Supplementary material for: In the eyes of the beholder: investigating the effect of visual probing on accuracy and gaze fixations when attending to facial expressions among primary and secondary callous-unemotional variants
Source: Eur Child Adolesc Psychiatry. 2019 Dec 6;29(10):1441–51. doi: 10.1007/s00787-019-01452-z (PMC7501133; doi:10.1007/s00787-019-01452-z)
Supplement: Supplementary file 1 — Supplementary material 1 (DOCX 19 kb) [file 787_2019_1452_MOESM1_ESM.docx]

Supplementary Material SM1. Emotion x probe x area interaction with fixation count as the dependent variable.
